# Supplementary material for: Identification of photoperiod-induced specific miRNAs in the adrenal glands of Sunite sheep (Ovis aries)
Source: Front Vet Sci. 2022 Jul 22;9:888207. doi: 10.3389/fvets.2022.888207 (PMC9354845; doi:10.3389/fvets.2022.888207)
Supplement: Supplementary file 1 [file Data_Sheet_1.ZIP › Supplementary file/miRDeep2 file.zip!/miRDeep2 file/Novel_Structure/SPLP42A2/Novel_657.pdf]

Provisional ID	:	NW_014642051.1_44748
Score total	:	2.7
Score for star read(s)	:	3.9
Score for read counts	:	-4.9
Score for mfe	:	2.1
Score for randfold	:	1.6
Score for cons. seed	:	
Total read count	:	2
Mature read count	:	1
Loop read count	:	0
Star read count	:	1

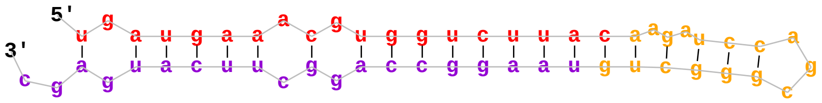

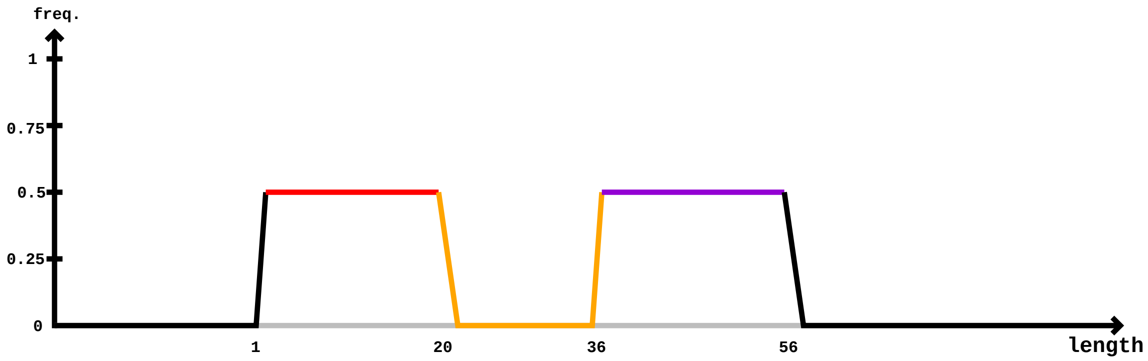

Mature Star

```
5'- uuuuccacuagagcacgggcugaugaaacguggucuuacaagauccagcgggcuguaaggccaggcuucaugagcucgagcucugaguuguuccagaagggaaacuuaa   -3'    obs
    uuuuccacuagagcacgggcugaugaaacguggucuuacaagauccagcgggcuguaaggccaggcuucaugagcucgagcucugaguuguuccagaagggaaacuuaa          exp
    .........(((((.((((((.(((((.(.((((((((((.(.(((...))))))))))))))).).))))).)))))).)))))(((((.((((....)))))))))..   reads      mm         sample
    ....................ugaugaaacguggucuuac..........................................................................   1          0          seq
    .......................................................uaaggccaggcuucaugagc..................................   1          0          seq
```
